# Noninvasive endoscopic hemostasis technique for post‐papillectomy bleeding using a novel self‐assembling peptide (with video)

**DOI:** 10.1002/jgh3.12853

**Published:** 2022-12-25

**Authors:** Akira Miyano, Takeshi Ogura, Hiroki Nishikawa

**Affiliations:** ^1^ 2nd Department of Internal Medicine Osaka Medical and Pharmaceutical University Osaka Japan

**Keywords:** bleeding, endoscopic sphincterotomy, endoscopic retrograde cholangiopancreatography, hemostasis, PuraStat

## Abstract

Although a novel hemostatic agent has been used for endoscopic mucosal resection in submucosal dissection, there are few case reports of its use in pancreato‐biliary endoscopic procedures. We describe a case of post‐endoscopic papillectomy bleeding in which endoscopic hemostasis was achieved using a novel hemostatic agent.

## Graphical abstract

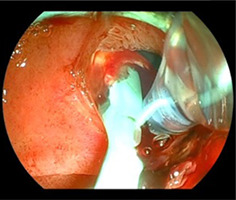

Endoscopic papillectomy (EP) has recently become the accepted procedure for early ampullary carcinoma as well as for ampullary adenoma. A recent large study, however, has reported post‐EP bleeding occurring with a frequency of 11%.^1^ To obtain hemostasis, methods such as coagulation are usually attempted, but these carry concerns such as perforation or post‐endoscopic retrograde cholangiopancreatography (ERCP) pancreatitis. A novel hemostatic agent (PuraStat gel, 3D Matrix Europe SAS, Caluire‐et‐Cuire, France) has recently become available. Although this gel has been used for endoscopic mucosal resection in submucosal dissection,^2^ there are few case reports of its use in pancreato‐biliary endoscopic procedures.^3^ We describe here a case of post‐EP bleeding in which endoscopic hemostasis was achieved using PuraStat.

A 79‐year‐old female was admitted for treatment of ampullary adenoma. An initial EP for this lesion was performed successfully, and a 7 Fr plastic stent was deployed for bile and pancreatic duct stenting without any adverse events, including bleeding. However, melena was observed 3 days later, and endoscopic examination was attempted. After introduction of the duodenoscope into the duodenum, blood clots and oozing were observed at the ampulla of Vater. As an ulcer had been observed during the papillectomy, we considered that using a traditional coagulation method might risk perforation, and so we selected the PuraStat injection technique accordingly. To prevent migration of the PuraStat into the third part of the duodenum, the duodenoscope was adjusted until the ampulla of Vater was at 3–6 O'clock position on the endoscopic image (Fig. 1). If PuraStat was applied under any other position, PuraStat itself may migrate into third part of the duodenum because of the gravity. The dedicated delivery system was then affixed around the bleeding point, PuraStat was applied, and hemostasis was achieved without any adverse events (Video S1). After 1 day, complete hemostasis was obtained.

**Figure 1:**
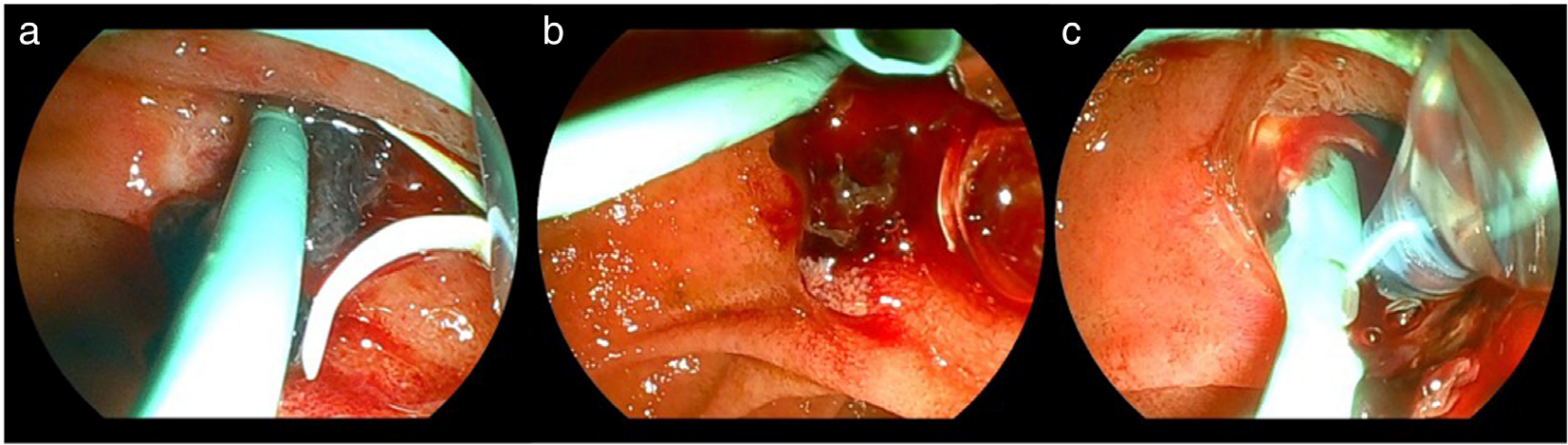
Ulcer formation is observed after papillectomy (a), and blood clots and oozing are observed at the ampulla of Vater (b and c). To prevent migration of PuraStat into the third part of the duodenum, the duodenoscope is adjusted until the ampulla of Vater is at 3–6 O'clock position in the endoscopic image.

In conclusion, PuraStat injection has no risk of perforation or pancreatitis and should thus be considered as a first‐line endoscopic hemostasis technique following post‐EP bleeding.

## Supporting information


**Video S1.** Blood clots and oozing are observed. To prevent migration of PuraStat dislocation into the third part of the duodenum, the duodenoscope is adjusted until the ampulla of Vater is at 3 to 6 O'clock position in the endoscopic image. PuraStat injection is performed, and endoscopic hemostasis is achieved.
